# Association of variant on the promoter of cluster of differentiation 74 in graves disease and graves ophthalmopathy

**DOI:** 10.1042/BSR20202072

**Published:** 2020-08-17

**Authors:** Yu-Huei Liu, Chiou-Yuan Shen, Fuu-Jen Tsai

**Affiliations:** 1 Graduate Institute of Integrated Medicine, China Medical University, Taichung, Taiwan; 2 Drug development center, China Medical University, Taichung, Taiwan; 3 Department of Medical Genetics and Medical Research, China Medical University Hospital, Taichung, Taiwan; 4 Department of Pediatrics, China Medical University Hospital, Taichung, Taiwan; 5 School of Chinese Medicine, China Medical University, Taichung, Taiwan

**Keywords:** Cluster of differentiation 74, Graves disease, Graves ophthalmopathy, single nucleotide polymorphisms, Subject Areas: Endocrinology, Immunology & Inflammation, Molecular Bases of Health & Disease

## Abstract

The macrophage migration inhibitory factor (MIF)/cluster of differentiation 74 (CD74) plays a role in immunological functions. The present study aims to investigate whether single-nucleotide polymorphisms (SNPs) in the *MIF* and *CD74* are risk factors for developing Graves ophthalmopathy (GO) in patients with Graves disease (GD). A case–control study enrolled 484 patients with GD (203 with and 281 without GO) and 1000 healthy individuals. SNPs were discriminated using real-time polymerase chain reaction. Hardy–Weinberg equilibrium, as well as frequencies of allele and genotype between GD patients with and without GO, were estimated using the Chi-square test. The effects of CD74 on adipocyte proliferation and differentiation were evaluated using 3T3-L1 preadipocytes. Quantitative DNA-immunoprecipitation was used to detect the binding capacity of NR3C1 and FOXP3 to A/G oligonucleotides. The results showed that individuals carrying the GG genotype at rs2569103 in the *CD74* had a decreased risk of developing GD (*P*=3.390 × 10^−11^, odds ratio (OR) = 0.021, 95% confidence interval (CI) = 0.003–0.154); however, patients with GD carrying the AG genotype at rs2569103 in the *CD74* had an increased risk of developing GO (*P*=0.009, OR = 1.707, 95% CI = 1.168–2.495). The knockdown of CD74 reduced adipocyte proliferation and differentiation. NR3C1 had a higher affinity for A, whereas FOXP3 had a higher affinity for G of rs2569103. The results suggested the existence of a link between the genetic variation of *CD74* promoter and the risk for developing GD and GO, which should be considered in clinical practice.

## Background

Graves disease (GD), a complex autoimmune disorder that occurs more often in women, is characterized by the presence of autoantibodies and thyroid-stimulating immunoglobulins, targeting the thyroid-stimulating hormone receptor to stimulate both thyroid hormone synthesis and thyroid gland growth, and results in hyperthyroidism and its accompanying features [1–3]. Graves ophthalmopathy (GO) is one common organ-specific complication affecting 25–50% of patients with GD [4]. Activation of orbital fibroblasts through proliferation and differentiation into adipocytes and myofibroblasts is thought to play a major role in the generation of the extracellular matrix. During inflammatory cell infiltration and edema, the activation augments the volume of tissues surrounding the eyes, which in turn leads to an increase in intraocular pressure [5].

Genetic predispositions, epigenetic regulations, and environmental factors are risk factors for GD and GO [6–10]. Representative studies shed new light on the pathogenesis of GD such as thyroid antigens, thyroid-stimulating hormone receptor, and human leukocyte antigen (*HLA*) class I and II regions [11,12]. However, the genome-wide approaches to determining the relative risks of developing GO are relatively limited [5,13]. Candidate gene approaches revealed that polymorphisms of genes involved in immune response and inflammation might be linked to the development of GO [5,6,14–21].

Cluster of differentiation 74 (CD74), encoded by *CD74*, is an HLA class II histocompatibility antigen gamma chain (also known as HLA-DR antigen-associated invariant chain) and a signal-transducing receptor of macrophage migration inhibitory factor (MIF) that maintains cell proliferation and survival [22,23]. The single-nucleotide polymorphisms (SNPs) in *HLA* class II and *MIF* play a role in the development of GD [24–26]. Conversely, the chromosome 5q31-33 region, where CD74 is located (5q32), may play a pivotal role in the development of GD and could be the susceptibility region for developing GD [27,28]. Results from mRNA-Seq also reveal CD74 as a novel signature for GD. However, to our knowledge, there is no study on the putative impact of *CD74* locus variations on the risk of GD or GO. In an attempt to contribute to the understanding of the pathogenic processes underlying GD and GO, a case–control study was designed to evaluate the association between SNPs in the upstream/downstream regulatory region of the MIF/CD74 axis and the risk of developing GD and GO.

## Methods

### Patients, healthy individuals, and DNA isolation

The study followed the Declaration of Helsinki and was approved by the Medical Ethics Committee of China Medical University Hospital (DMR100-IRB-144, CMUH103-REC2-071). A total of 484 patients with GD (384 females/100 males; mean age 39.6 y; range 13.9–83.9 y at enrollment) from the China Medical University Hospital, and 203 patients had GO and 281 did not. All participants provided written informed consent. Detailed descriptions of the inclusion/exclusion criteria, blood drawing and handling, genomic DNA storage, and quality assurance have been described [15,17]. SNP data for 1000 ethnicity-matched healthy individuals were obtained from the Taiwan biobank.

### SNP selection and genotyping

SNPs were selected based on the following criteria: (i) a threshold minor allele frequency (MAF) in the Asian population of 0.10; (ii) primer/probe set passed by the manufacturer criteria to ensure a high genotyping success rate; and (iii) SNP data for healthy individuals could be obtained without imputation from the Taiwan biobank. Four SNPs, namely, rs476240 and rs507715 in the downstream region of *MIF* (which is also the upstream region of MIF antisense RNA 1 [*MIF-AS1*]), as well as rs13175409 and rs2569103 in the upstream region of *CD74*, were analyzed. Genotyping using specific primer/probe sets have been described previously [15,17].

### Cell culture

The human HEK293 cells and mouse 3T3-L1 preadipocytes were obtained from Bioresource Collection and Research Center (BCRC, Hsinchu, Taiwan) and maintained in Dulbecco’s modified Eagle’s medium (DMEM, 12100046, Thermo Fisher Scientific, Waltham, MA, U.S.A.) with 10% fetal bovine serum (16000044), 50 U/ml penicillin and 50 μg/ml streptomycin (15070063), and 2 mM L-glutamine (25030081) at 37°C in a humidified atmosphere of 5% CO_2_.

### CD74 knockdown

Short hairpin RNAs (shRNAs) obtained from the RNAi core (Academia Sinica, Taipei, Taiwan) were used in CD74 knockdown experiments. For CD74 knockdown, confluent 3T3-L1 preadipocytes in six-well dishes were incubated in Opti-MEM (Thermo Fisher Scientific) and transfected with either CD74 shRNA or nonspecific shRNA using Lipofectamine 3000 (Thermo Fisher Scientific) according to the manufacturer’s protocol. After 6 h, the medium was replaced with complete DMEM with a differentiation cocktail (500 μM 3-isobutyl-1-methylxanthine, 1 μM dexamethasone, and 10 μM insulin) to induce differentiation into mature adipocytes (day 0).

### Western blotting

Equal amounts of protein lysates were subjected to sodium dodecyl sulfate-polyacrylamide gel electrophoresis and then transferred to polyvinylidene fluoride membranes. After blocking with 5% skim milk, the membranes were incubated with primary antibodies and subsequently with appropriate peroxidase-conjugated secondary antibodies. Primary antibodies, including targets, catalog numbers, dilutions, and suppliers, were as follows: antibodies specific to CD74 (GTX110477, 1:500) were from GeneTex, Hsinchu, Taiwan and antibodies specific to actin (MAB1501, 1:5000) were from MilliporeSigma, St. Louis, MI, U.S.A.

### Adipocyte differentiation

The 2-day post-confluency preadipocytes were cultured in complete DMEM with a differentiation cocktail (500 μM 3-isobutyl-1-methylxanthine, 1 μM dexamethasone, and 10 μM insulin). On day 3 of differentiation, cells were switched to complete DMEM with 10 μM insulin for the remaining duration of differentiation.

### Cell counting

3T3-L1 cells were detached from six-well plates using 0.25% trypsin (Thermo Fisher Scientific), resuspended in complete DMEM, and counted using a cell counter (Millipore) every day from day 0–4.

### Oil Red O staining

Differentiated adipocytes were fixed in 10% formalin and stained for 30 min with Oil Red O (MilliporeSigma) working solution (0.4% Oil Red O dye in 60% isopropanol). Oil Red O was extracted using 100% isopropanol, and the absorbance was measured at 540 nm using a spectrophotometer.

### Cell culture and extraction of nuclear proteins from established NR3C1, FOXP3, and CD74 transformants

Cells were transfected with the pCMV3−C−Myc−NR3C1, pCMV3−C−Myc−FOXP3, or pCDNA4-CD74 using the Lipofectamine 3000 kit (Thermo Fisher Scientific) according to the manufacturer’s protocol. The nuclear proteins were extracted using NE-PER nuclear and cytoplasmic extraction reagents (Thermo Fisher Scientific) supplemented with protease inhibitor cocktail and phosphatase inhibitors (Roche, Basel, Switzerland) according to the manufacturer’s protocol.

### Quantitative DNA immunoprecipitation (qDNA-IP) assay

qDNA–IP assays were performed on nuclear extracts from established FOXP3 and NR3C1 transformants. DNA binding of FOXP3 or NR3C1 was assessed using the annealed double strand oligonucleotides: 5′-biotin-labeled rs2569103A probes 5′-CCAAATGGCTGGTTTCAGGGCTGGAGATGGGGG-3′ and 5′-CCCCCATCTCCAGCCCTGAAACCAGCCATTTGG-3′, as well as 5′−biotin-labeled rs2569103G probes 5′-CCAAATGGCTGGTTTCGGGGCTGGAGATGGGGG-3′ and 5′−CCCCCATCTCCAGCCCCGAAACCAGCCATTTGG−3′ (PURIGO Biotechnology, Taipei, Taiwan). For the binding reactions, 5 μg of nuclear proteins were incubated with or without labeled oligonucleotides in binding buffer [50 mM Tris–HCl (pH 7.5), 250 mM NaCl, 5 mM MgCl_2_, 2.5 mM EDTA, 2.5 mM DTT, 0.25 mg/ml poly(dI–dC), and 20% glycerol] for 30 min at 25°C in a final volume of 20 μl. FOXP3– or NR3C1–nucleotide complexes were cross-linked with formaldehyde (1% final concentration) for 10 min at room temperature, followed by immunoprecipitation with antibodies specific to Myc tag (GTX115046, 1:100, GeneTex) and Protein A/G magnetic beads (GE Healthcare). Immunoprecipitated DNA was detected using horseradish peroxidase-conjugated streptavidin. The reaction was developed with the 3,3′,5,5′-tetramethylbenzidine reagent (Sigma) and read at 450 nm with a Microplate reader (BioRad, Hercules, CA, U.S.A.).

### Statistical analyses

The statistical analyses were performed using the PASW Statistics 18.0 software from IBM (Armonk, NY, U.S.A.). A *t*-test was used to evaluate the associations between GO and age. A Chi-square test was used to evaluate the associations between polymorphisms and GD or GO. Screening for linkage disequilibrium (LD) was performed using Haploview ver. 4.1 [29]. A two-tailed *P*-value less than 0.05 with Bonferroni correction (0.05/4) was considered statistically significant [30]. Logistic regression with a 95% confidence interval (CI) was used to estimate odds ratios (ORs).

## Results

### Demographic data, clinical characteristics, and their correlations with GO in patients with GD

The frequency distributions of clinical characteristics, such as goiter, nodular hyperplasia, myxedema, vitiligo, and age, in male and female groups were compared between the patients with GD with or without GO. As demonstrated in Table 1, gender and age were significantly associated with GO in patients with GD. Even myxedema was associated with GO in patients with GD; however, due to a limited number of cases, the association needs further investigation. These results adhered to other epidemiological results that GO occurred more commonly in the middle-aged female population.

**Table 1:** Demographic data and clinical characteristics of graves disease patients with or without graves ophthalmopathy

Characteristic	GD/non-GO, *N* (%)	GD/GO, *N* (%)	*P*
Number of patients	281 (58.1)	203 (41.9)	
Female gender	232 (82.6)	152 (74.9)	0.039^a^*
Age of diagnosis (Year) (Mean ± SD) [Range]	41.4 ± 12.7 [13.9−83.9]	37.0 ± 10.7 [18.0−71.9]	5.802 × 10^-5^^b^ ***
Presence of goiter			0.165^a^
No	18 (6.4)	14 (6.9)	
1a	21 (7.5)	5 (2.5)	
1b	32 (11.4)	22 (10.8)	
2	177 (63.0)	132 (65.0)	
3	33 (11.7)	30 (14.8)	
Presence of nodular hyperplasia	27 (9.6)	23 (11.3)	0.539^a^
Presence of myxedema	1 (0.4)	5 (2.5)	0.039^a^ *
Presence of vitiligo	2 (0.7)	2 (1.0)	0.743^a^
With radioiodine therapy history	8 (3.3)	10 (5.5)	0.273^a^
With thyroid surgery history	27 (11.2)	14 (7.7)	0.227^a^
With smoke history	52 (21.6)	44 (24.2)	0.527^a^
Free T3 (pg/ml)	5.3 ± 4.8	5.4 ± 5.0	0.900^a^
Free T4 (ng/dl)	1.7 ± 1.3	1.8 ± 1.4	0.692^a^
T3 (ng/dl)	218.7 ± 169.8	183.2 ± 119.8	0.146^a^
T4 (μg/dl)	10.4 ± 6.3	9.1 ± 6.3	0.310^a^
TSH (μIU/ml)	1.9 ± 7.9	2.5 ± 9.2	0.479^a^
TRAb positive (%)	40.0 ± 25.7	42.4 ± 25.5	0.482^a^

Abbreviations: GD, graves disease; GO, graves ophthalmopathy; *N*, number.

a Frequencies of genotypes were determined by the chi-square test using 2 × 2 or 2 × 5 contingency tables.

b Significance of age were evaluated by *t* test.

* *P*<0.05

*** *P*<0.001.

### LD among SNPs of MIF and CD74

**Figure 1:**
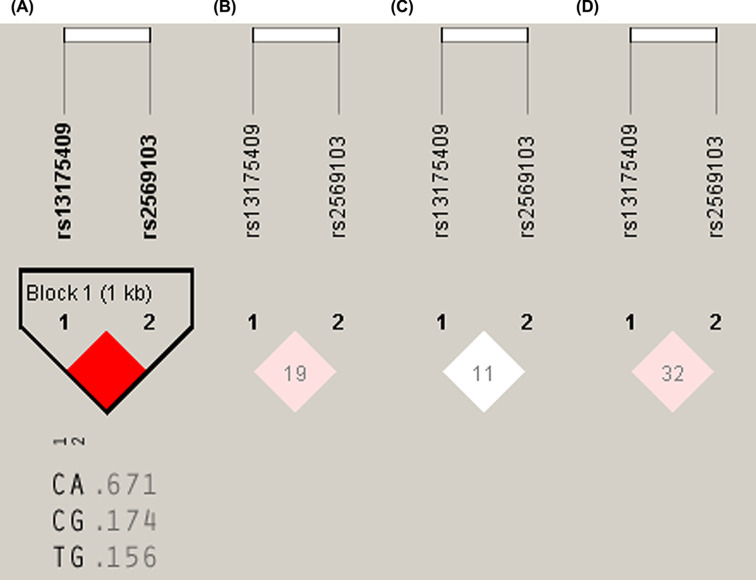
Linkage disequilibrium (LD) values between the two polymorphisms, rs13175409 and rs2569103, in the CD74 region in a Taiwanese-Chinese population The color scale reflects the strength of LD between the two single nucleotide polymorphisms (SNPs). (**A**) Healthy individuals. (**B**) Patients with Graves disease (GD), with and without Graves ophthalmopathy (GO). (**C**) Patients with GD without GO. (**D**) Patients with GD with GO.

Four SNPs of the *MIF* and *CD74* were genotyped to determine whether polymorphisms in these genes influence the development of GO in patients with GD. The distribution of the four SNPs fit the Hardy–Weinberg equilibrium (HWE) in patients with GD and healthy individuals. However, the strong (*r*^2^>0.8) LD *r*^2^ values calculated for the two SNPs at the *CD74* in healthy individuals were not observed in patients with GD, with or without GO, suggesting that there is more variation in the extent of LD within CD74 in patients with GD (Figure 1).

### Allele and genotype distributions of CD74 contribute to GD/GO development

No significant association was found in the examined SNPs of *MIF*, nor was a significant association found between the polymorphisms and the clinical features or the indicators of thyroid function, including free triiodothyronine (T3), free thyroxine (T4), thyroid stimulating hormone (TSH), and thyrotropin receptor antibodies (TRAbs), in patients with GD. However, allele frequencies showed that individuals carrying a G allele at rs2569103 in the *CD74* had a reduced risk of developing GD (*P*=0.005, OR = 0.785, 95% CI = 0.663–0.929) (Table 2). Genotype frequencies further showed that individuals carrying the GG genotype at rs2569103 in the *CD74* had a reduced risk of developing GD (*P*=3.390 × 10^−11^, OR = 0.021, 95% CI = 0.003−0.154), which was consistent with results from allele frequencies; however, the patients with GD carrying the AG genotype at rs2569103 in the *CD74* had an increased risk of developing GO (*P*=0.009, OR = 1.707, 95% CI = 1.168−2.495) (Table 3).

**Table 2:** Allele distributions of *MIF* and *CD74*

Genotypes	Control, *N* (%)	GD/non-GO, *N* (%)	GD/GO, *N* (%)	Control vs GD, *P*^a^	Control vs GD, OR (95%CI)	Non-GO vs GO, *P*^a^	Non-GO vs GO, OR (95%CI)
*MIF* rs476240							
A	270 (13.5)	79 (14.1)	53 (13.1)	0.919		0.654	
G	1730 (86.5)	483 (85.9)	353 (86.9)				
*MIF* rs507715							
A	738 (36.9)	234 (41.6)	156 (38.4)	0.075		0.314	
C	1262 (63.1)	328 (58.4)	250 (61.6)				
*CD74* rs13175409							
C	1689 (84.5)	479 (85.2)	354 (87.2)	0.252		0.385	
T	311 (15.6)	83 (14.8)	52 (12.8)				
*CD74* rs2569103							
A	1342 (67.1)	422 (75.1)	277 (68.2)	0.005*	1	0.019	
G	658 (32.9)	140 (24.9)	129 (31.8)		0.785 (0.663−0.929)^b^#		

Abbreviations: CI, confidence interval; GD, graves disease; GO, graves ophthalmopathy; *N*, number; OR, odds ratios.

a Frequencies of genotypes were determined by the chi-square test using 2 × 2 or 2 × 3 contingency tables.

b Odds ratios and 95% CI per genotype were estimated by applying unconditional logistic regression.

* *P*<0.05 with Bonferroni correction; #, OR with significance.

**Table 3:** Genotype distributions of *MIF* and *CD74*

Genotypes	Control, *N* (%)	GD/non-GO, *N* (%)	GD/GO, *N* (%)	Control vs GD, *P* ^a^	Control vs GD, OR (95%CI)	Non-GO vs GO, *P* ^a^	Non-GO vs GO, OR (95%CI)
*MIF* rs476240							
AA	17 (1.7)	5 (1.8)	6 (3.0)	0.713		0.394	
AG	236 (23.6)	69 (24.6)	41 (20.2)				
GG	747 (74.7)	207 (73.7)	156 (76.8)				
*MIF* rs507715							
AA	138 (13.8)	52 (18.5)	33 (16.3)	0.144		0.609	
AC	462 (46.2)	130 (46.3)	90 (44.3)				
CC	400 (40.0)	99 (35.2)	80 (39.4)				
*CD74* rs13175409							
CC	712 (71.2)	205 (73.0)	152 (74.9)	0.494		0.234	
CT	265 (26.5)	69 (24.6)	50 (24.6)				
TT	23 (2.3)	7 (2.5)	1 (0.5)				
*CD74* rs2569103							
AA	437 (43.7)	141 (50.2)	75 (36.9)	3.390 × 10^-11^*	1	0.009*	1
AG	468 (46.8)	140 (49.8)	127 (62.6)		1.154 (0.925−1.441) ^b^		1.705 (1.179−2.467)^b^^#^
1.707 (1.168−2.495)^c^^#^							
GG	95 (9.5)	0 (0.0)	1 (0.5)		0.021 (0.003−0.154)^b^^#^		0.000−

Abbreviations: CI, confidence interval; GD, graves disease; GO, graves ophthalmopathy; *N*, number; OR, odds ratios.

a Frequencies of genotypes were determined by the chi-square test using 2 × 2 or 2 × 3 contingency tables.

b OR and 95% CI per genotype were estimated by applying unconditional logistic regression.

c OR and 95% CI per genotype were estimated by adjusting with gender, age, and myxedema.

* *P*<0.05 with Bonferroni correction

# OR with significance.

### Knockdown of the expression of CD74 inhibits 3T3-L1 adipocyte differentiation

**Figure 2:**
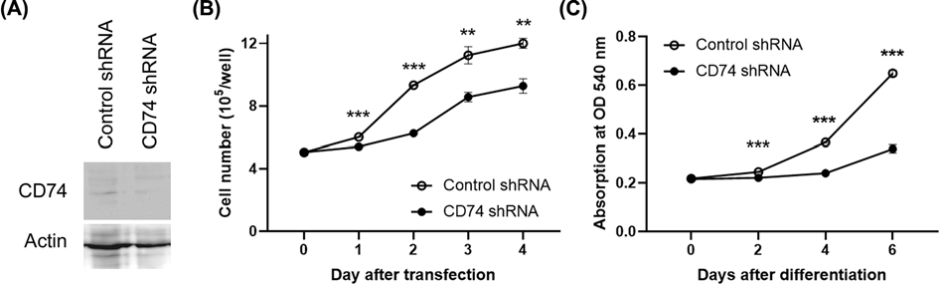
Changes in adipocyte differentiation and proliferation after knockdown of CD74 (**A**) Endogenous expression of CD74 protein in 3T3-L1 cells was examined, and knockdown of CD74 was examined by Western blotting. Actin was used as an internal control. (**B**) The down-regulation of CD74 inhibits cell growth. 3T3-L1 cells were detached from six-well plates and counted. ** P<0.01, *** P<0.001 CD74 knockdown vs control cells. (**C**) Cells were stained with Oil Red O after inducing differentiation. Quantitative analyses were performed by measurement of optical density (OD) at 540 nm in extracts from Oil Red O-stained cells transfected with CD74 short hairpin RNA (shRNA) and control shRNA. ***P<0.001 CD74 knockdown vs control cells.

The swelling of extraocular orbital fat is one reason that the development of GO is triggered [31]. To understand the possible regulation between CD74 and adipocyte differentiation, 3T3-L1 cells were chosen as an experimental model. The expression of CD74 in CD74 knockdown (CD74-KD) cells by shRNA was confirmed as compared with those with control of shRNA (Figure 2A). Cell numbers of CD74-KD and control cells were counted every day. The knockdown of CD74 decreased cell proliferation from 1–4 days after induction (Figure 2B). In addition, the degree of Oil Red O staining was weaker in CD74-KD cells than in control cells (65.7% on day 4 and 52.3% on day 6, respectively, for CD74 shRNA vs control cells) (Figure 2C).

### Different binding affinities of NR3C1 and FOXP3 for CD74 promoter depends on SNP rs2569103

**Figure 3:**
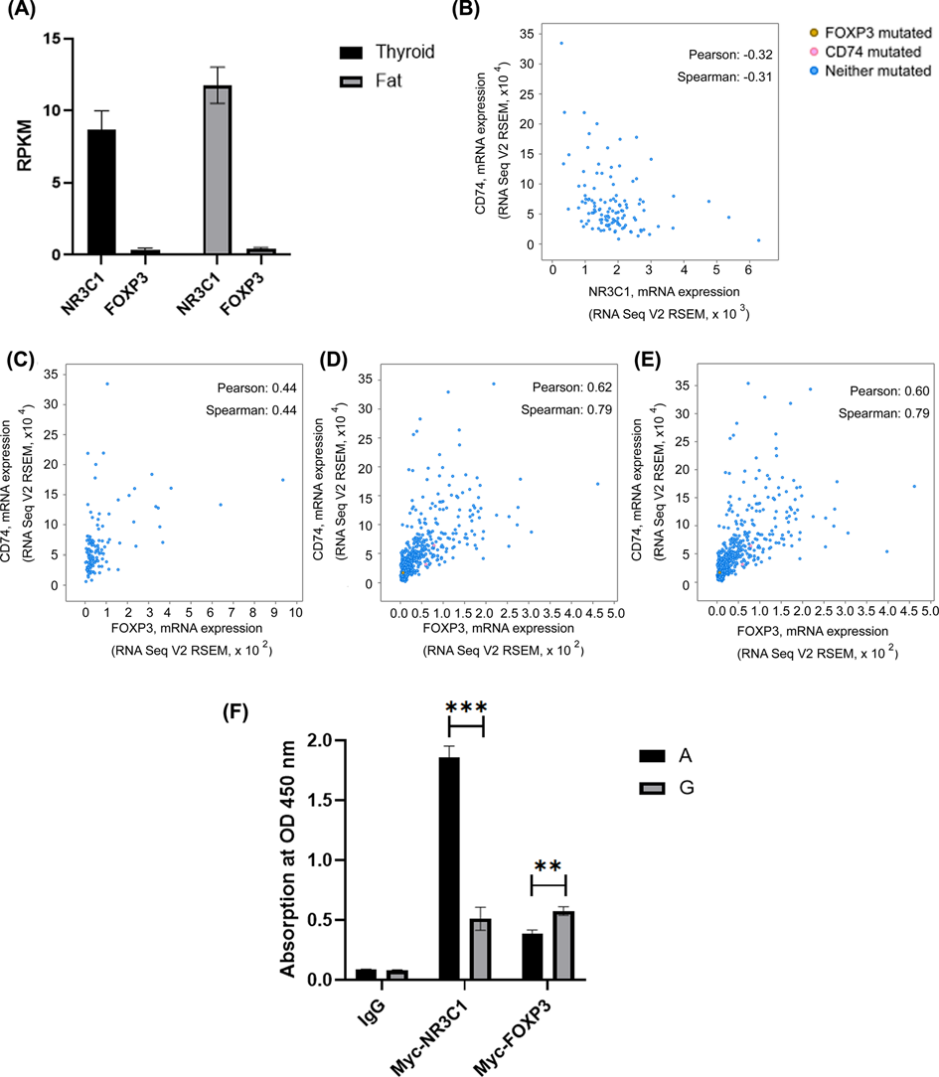
Different binding affinities of NR3C1 and FOXP3 for CD74 promoter depends on single-nucleotide polymorphism (SNP) rs2569103 (**A**) RNA-seq analysis of NR3C1 and FOXP3 in thyroid and fat tissues from public datasets (PRJEB4337). (**B**–**E**) Bioinformatic analysis of mRNA expression correlation between NR3C1 and CD74 or FOXP3 and CD74. The mRNA expression of NR3C1 and CD74 in thymoma samples (B); and the mRNA expression of FOXP3 and CD74 in thymoma samples (C), well-differentiated papillary thyroid carcinoma (D), and well-differentiated thyroid cancer (E). (**F**) Probe with promoter sequence containing rs2569103 (probe A) has a higher affinity for NR3C1, whereas G at rs2569103 (probe G) has a higher affinity for FOXP3 as shown by quantitative DNA immunoprecipitation (qDNA-IP) assay. ** P<0.01, *** P<0.001 probe A vs probe G.

The *CD74* SNP rs2569103 was located within the upstream region of *CD74* and showed the strongest association with the disease, making it a possible target for transcription factors. Indeed, the putative transcription factor-binding sites were predicted using PROMO [32,33]. At SNP rs2569103, the A allele generates motifs for nuclear receptor subfamily 3, group C, member 1 (NR3C1) (TC**A**GG), whereas the G allele generates a motif for forkhead box P3 (FOXP3) (GTTTC**G**). Bulk RNA-seq analysis of NR3C1 and FOXP3 in thyroid and fat tissues from public datasets (PRJEB4337) were demonstrated (Figure 3A). To interpret the possible regulatory mechanisms of these molecules, published mRNA expression results were explored. The mRNA expression of NR3C1 only showed a negative correlation with that of CD74 in thymoma samples (Pearson’s correlation: −0.32, Spearman’s correlation: −0.31) (Figure 3B), whereas the mRNA expression of FOXP3 showed a positive correlation with that of CD74 (Pearson’s correlation: 0.44, 0.62, 0.60; Spearman’s correlation: 0.44, 0.79, 0.79 in thymoma samples, well-differentiated papillary thyroid carcinoma, and well-differentiated thyroid cancer, respectively) (Figure 3C–E). The qDNA-IP results supported that NR3C1 tends to bind to probes with promoter sequence containing AA at rs2569103, whereas FOXP3 tends to bind to probes with promoter sequence containing GG at rs2569103 (Figure 3F). These results suggested that the CD74 expression may be orchestrated by complex transcription factor networks. The AA genotype may play a role in response to NR3C1-induced CD74 downregulation, whereas the GG genotype on rs2569103 on the *CD74* promoter may play an additional role in response to FOXP3-induced CD74 up-regulation.

## Discussion

Environmental factors and genetic loci have been thought to be associated with immune regulation [8,10]. Here we identified new candidates, *CD74* alleles and genotypes, for the susceptibility of GD and GO in a Taiwanese-Chinese population. CD74 is involved in adipocyte differentiation through its differential promoter binding affinity for transcription factors. To the best of our knowledge, this is the first study to demonstrate novel *CD74* polymorphisms in association with the development of GD and GO. Our results support whole-genome screening studies in that the chromosome 5q32 may play a role in generating GD and GO in humans.

The thyroid gland of patients with GD revealed marked enlargement of the gland due to autoantibodies. Patients with accompanying GO exhibited enlargement of the retro-orbital connective tissue and extraocular muscles, in part due to the inflammatory deposition of glycosaminoglycans, collagen, and fat [34]. Indeed, genes involved in the regulation of cell survival, DNA transcription, and protein synthesis have been considered risk factors for GD and GO [10,35]. Overexpression of CD74 plays a crucial role in preventing hyperreactivity between immature antigens and major histocompatibility complex class II as well as cell growth and survival, whereas down-regulation of CD74 is often correlated with autoimmunity and cell apoptosis [36]. Upon expression of surface CD74, the cells may transduce survival signaling through extracellular signal-regulated kinase 1/2 or c-Jun N-terminal kinase (JNK) mitogen-activated protein kinase (MAPK) pathways or AKT pathways in a MIF-dependent manner, thereby improving cell survival and proliferation [23,37]. Due to the limitation to find identical cells expressed GG or AA genotype on rs2569103, current results we did not show the direct impact of these transcription factors to the CD74 expression. Further evidence such as RNA-seq as secondary data was warranted. The results showed that GD patients with or without GO, although loss the protective GG genotype, most of them hold AG heterogenous genotype instead, suggested the loss-of-protect effect on the disease. In the present study, cell-based experiments showed that CD74 is involved in adipocyte differentiation, but the link toward GO development remained to be investigated. On the other hand, the GG genotype on rs2569103, with a higher frequency in healthy individuals (Table 3), increased the binding of FOXP3 to the *CD74* promoter (Figure 3F), thereby increasing CD74 up-regulation and protecting autoimmune responses. Conversely, the AA genotype on rs2569103 increases the binding of NR3C1 to the *CD74* promoter, which down-regulates CD74 and increases autoimmune response and manifestations of GD/GO. Due to the limitation to find identical cells expressed GG or AA genotype on rs2569103, current results we did not show the direct impact of these transcription factors to the CD74 expression. Further evidence such as RNA-seq as secondary data was warranted. The results showed that GD patients with or without GO, although they lost the protective genotype, most of them hold the AG heterogenous genotype instead, suggesting the loss-of-protection effect of the disease. Further studies on the detailed mechanisms through CD74-derived adipocyte differentiation are warranted.

Conversely, the ligand of CD74, MIF, has previously been reported to be counter-regulatory to glucocorticoid secretion [36–38]. The glucocorticoid-induced MIF secretion was noted at 180 min after dexamethasone administration [39]. In addition, nonsteroidal anti-inflammatory drugs, such as aspirin, ibuprofen, and naproxen, have been used to relieve the pain and inflammation of GO. This evidence further supports the crucial role of CD74 in the transduction of MIF signaling. However, due to the limited population of the minor polymorphism, the present study is unable to reach the interactions among cells and molecules in the orbital microenvironment and their association toward the target polymorphism due to the inaccessibility of the orbital tissues. The current finding may have further implications for understanding the link between the polymorphism/expression of CD74 and current treatments for GO—a therapeutic effect issue that might be of value for future treatment strategies targeting MIF or CD74.

In conclusion, the current study identified new SNPs in the *CD74* that were found to be associated with GD and GO in a Taiwanese-Chinese population. Biological studies provide insights into the genetic information that influences the development of GD and GO via adipocyte proliferation and differentiation.

## Perspectives

- The impact of genetic factors on the orbital microenvironment cannot be closely monitored due to the inaccessibility of the orbital tissue. Studies on feasible cell-based models may help elucidate how genetic factors such as CD74 SNPs modulate the target gene expression.
- The present study combined clinical observations and cell models to investigate how CD74 polymorphisms affect adipocyte proliferation and differentiation.
- The present clinical observations suggest that the genetic factors of CD74 should be considered in clinical practice.

## Abbreviations

CD74: cluster of differentiation 74
CI: confidence interval
FOXP3: forkhead box P3
GD: graves disease
GO: graves ophthalmopathy
HLA: human leukocyte antigen
HWE: Hardy–Weinberg equilibrium
JNK: c-Jun N-terminal kinase
LD: linkage disequilibrium
MAPK: mitogen-activated protein kinase
MIF: macrophage migration inhibitory factor
NR3C1: nuclear receptor subfamily 3, group C, member 1
OR: odds ratio
PCR: polymerase chain reaction
qDNA-IP: quantitative DNA immunoprecipitation
SNP: single-nucleotide polymorphism
T3: triiodothyronine
T4: free thyroxine
TRAb: thyrotropin receptor antibody
TSH: thyroid stimulating hormone
